# Goals of cure: Perspectives on the concept of cure in type 2 diabetes

**DOI:** 10.1111/jep.13666

**Published:** 2022-02-12

**Authors:** David Zhao

**Affiliations:** ^1^ Department of Medicine Johns Hopkins University School of Medicine Baltimore Maryland USA

**Keywords:** bariatric surgery, cure, goals of care, medical uncertainty, remission, type 2 diabetes

## Abstract

**Rationale, Aims and Objectives:**

Type 2 diabetes (T2D) is an archetypical chronic condition of significant prevalence. Yet the concept of cure in the context of T2D reveals an interplay between the medical imagination and clinical realities that can shift the course of a patient's care. There are two domains in which cure is sociologically constructed: the professional domain occupied by clinicians treating people with T2D, and the lay domain occupied by T2D patients. Lay epistemologies of cure tend to be focused on modifying the experience of having T2D, while professional epistemologies tend to focus on modifying the disease through medical treatment. The objective of this study is to explore the role of the concept of cure in the context of type 2 diabetes, a model for chronic disease.

**Methods:**

Through surveys and interviews of T2D patients, providers and researchers at an urban academic medical centre, I explore the perspectives and attitudes each group have towards the concept of cure in T2D. Semi‐structured interviews of T2D professionals and patient surveys consisting of free response questions and Likert scale items were thematically analysed for perspectives on cure in T2D.

**Results:**

Sixteen T2D patients met inclusion criteria and consented to the survey and ten T2D professionals were interviewed. Cure is conceived of heterogeneously both within and between epistemologies. Patients carry hopes of cure predicated on eliminating the unpleasant experiences of T2D and its treatments, while T2D professionals tend to avoid invoking the concept of cure, at least to patients, on grounds of clinical uncertainty. However, the concept of cure is a significant motivator of treatment in both lay and professional epistemologies.

**Conclusion:**

Different viewpoints on cure in T2D present an opportunity for shared meaning and decision making between patients and their providers that can frame the best possible outcome for patient care.

## 1 INTRODUCTION

Type 2 diabetes (T2D), which impacts an estimated 463 million people globally,^1^ presents a formidable medical challenge to patients, healthcare professionals and policymakers. Given its prevalence, T2D can be considered an index condition for chronic disease, the archetype of a condition that can only be managed not cured. Still, the idea of cure manifests in the medical imagination of T2D patients and their providers. A paradox exists between the categorical notion of a cure and the variable spectrum of a chronic condition. It is not surprising that the desire to cure exists, but it is worth examining how it takes shape in the context of chronic care. What is the role of the concept and the discussion of cure for T2D, the model of long‐term management of a chronic condition?

The literature shows differing attitudes towards cure in T2D both between and within T2D professionals and patients. This depends on the understanding of what T2D is, or ontology of T2D, which will determine how one knows T2D is cured, or epistemology of cure. In the professional domain, where T2D is clinically diagnosed based on techniques consistent with the biomedical model of medicine, the curability of T2D is disputed. In the clinical landscape of T2D, the technical editor of *Diabetic Medicine* C. Fox notes that, ‘the word “cure” generates strong negative feelings in some professionals working in the field, because they doubt the concept of a cure for this condition’.^2^ In 2009, a group of endocrinology, diabetes education, transplantation, metabolism, bariatric/metabolic surgery and haematology‐oncology professionals came together to discuss the differences between management, remission and cure in T2D, and whether it is accurate to describe any chronic condition as curable.^3^ While no consensus was reached, many stopped short of calling T2D curable. Instead using ‘prolonged remission’, a term often used in cancer, was viewed by some to be more appropriate to illustrate the constant underlying physiologic tendency towards a diabetic state once it has been diagnosed. They defined prolonged remission as hyperglycaemia below diagnostic thresholds for diabetes and no active pharmacologic therapy or operations for the condition for at least 5 years.^3^ Because the ontology of T2D is based on a biomedical model of medicine, the epistemology of cure is derived from a molecular pathophysiology, and thus cure must address this level of existence of T2D; something many clinicians do not believe is yet guaranteed.

However, in the field of bariatric surgery, cure for T2D is discussed in the context of a surgical approach to treating T2D, an anatomical rather than molecular modification of the cause of T2D. An outcome frequently reported with bariatric surgery is apparent resolution of T2D, or removing the need for T2D treatment, at a rate of up to 80%.^4^, ^5^, ^6^ Bariatric surgery is cited by professionals in the field as a potential specific cure for T2D because markers of T2D remission are detected before weight loss, suggesting that the procedure addresses underlying causes of T2D independently of weight loss.^7^, ^8^, ^9^, ^10^, ^11^ More precisely, ‘complete T2DM remission is defined as fasting plasma glucose <100 mg/dl and/or HbA1c < 6% for at least 1 year after surgery in the absence of glucose‐lowering pharmacologic treatment…A prolonged complete T2DM remission, extending beyond 5 years, may be viewed as operationally equivalent to cure’.^7^ Therefore, the bariatric surgery perspective is an example of heterogeneous conceptions of T2D cure even within the professional domain.

The curability of T2D is a nontrivial problem for T2D patients, who may largely rely on a lay epistemology of cure based on an ontology rooted in the personal experience of having T2D. T2D, how it came to be and its consequences, is largely personalized in the lay domain, such as through experiencing individual stress rather than describing a molecular pathophysiology.^12^ This could result in the development of personalized strategies to manage T2D independent of clinical management, for example, coping through religious beliefs or normalising their condition with actions that help them feel well.^13^ Each T2D patient has a personal narrative of how they got T2D, and thus have a personal conception of cure. The socio‐cultural context can also influence an individual's conception of T2D and cure outside of the healthcare system.^14^

No matter how a person developed T2D, having it cured is an important goal and there is some flexibility with how that may be achieved. A survey distributed to T2D patients found that the highest priority question is: ‘Can T2D be cured or reversed, what is the best way to achieve this and is there a point beyond which the condition cannot be reversed?’.^15^ Furthermore, patients most want to see T2D research conducted towards ‘managing T2D with comorbidities, controlling blood sugar levels, finding a cure and understanding causes of T2D’.^16^ When T2D patients were interviewed after a weight loss intervention study,^17^ four themes emerged to form a patient narrative of cure for T2D. The results were:

1. ‘Building behavioural autonomy’ as a process of growing confidence to engage in health behaviours that are independent of those of other people.
2. ‘Behavioural contagion’ describing how one's new health behaviours tend to affect those of other people.
3. ‘From rigid to flexible restraint’, reflecting the changes in attitudes and behaviours required for a successful adaptation from weight loss to weight maintenance.
4. ‘Shift in identity’, representing changes in the participants' perceptions of themselves.

These themes appear independent from the clinical and technical discussions of cure in T2D in the professional domain and support an internal locus of control for patients. Behavioural autonomy suggests that an aspect of cure involves independence from the healthcare system and the ability to make healthy choices through self‐care.^18^ Behavioural contagion points towards the significant involvement of the popular and folk domains of health care, such as cooking healthy meals with family members. Flexible restraint, though recognising that there is still some degree of management involved post‐diabetes, is perhaps the theme most agreeable with clinical descriptions of diabetes resolution. Finally, a shift in identity places an emphasis on the importance of a patient's own self‐affirmed determination of cure. As Canguilhem identified, a cure to the patient is reclaiming one's life.^19^ As such, one's personal experiences and circumstances form the basis of their health beliefs and the ways cure may be achieved, which may not be in line with the biomedical conceptualisation of T2D and cure.^20^

This qualitative study evaluates the attitudes of T2D patients and the providers that treat them and do research on their condition at an urban academic medical centre. The aim of this study is to clarify different perspectives about cure in the chronic condition of T2D and to identify commonalities that may help facilitate clearer communication about goals of care for T2D.

## 2 METHODS

### 2.1 Study design, setting and populations

This qualitative study was approved by the University of Chicago Biological Sciences Division Institutional Review Board. Participants were recruited and interviewed or surveyed from January to March 2020. It was explained to participants that they were being surveyed about their goals of care for T2D and experience living with T2D.

The study had two arms, one for recruiting T2D patients to fill out a survey and the other for interviewing T2D clinicians and researchers. In the patient arm, patients were recruited from the clinic population at the Kovler Diabetes Center at the University of Chicago Medical Center following an endocrinology appointment from five different providers once a week for 3 months. Potential participants were screened by their provider to participate in a survey and then were formally consented after their appointment. The inclusion criteria were adults 18 years or older with a diagnosis of T2D and actively undergoing T2D treatment. Demographic data such as race and gender were not collected as the goal of this study was to characterize the personal experiences of T2D patients rather than to analyze demographic differences regarding those experiences. The survey is based on questions from the *Diabetes Care Profile*,^21^ modified to focus on patient perceptions of cure and their interactions with their providers on that topic. The survey was reviewed by a T2D expert, anthropologist and philosophy of science faculty.

T2D clinicians and researchers at the University of Chicago were recruited through email for an approximately 30‐min interview and an effort was made to include a diverse selection of T2D experts. The specialisations of faculty interviewed ranged from endocrinology, biomedical research, bariatric surgery and weight management. In semi‐structured interviews using a grounded theory approach to develop a theory of T2D cure, participants were asked questions on three themes: (1) about their perspective on the curability of T2D, (2) how a cure could be achieved and (3) communicating information about T2D treatment to patients. The interview guide is provided in the appendix.

### 2.2 Data collection

Patients were asked if they were willing to fill out a survey asking about their views and experiences with type 2 diabetes care. If the patient agreed to participate, they filled out anonymous surveys at a quiet library adjacent to the Kovler Center on paper or at their own convenience through an identical online Qualtrics survey. Patients were instructed to fill out as much of the survey as they wanted and incomplete surveys were included in the study. The survey consisted of four free‐response questions asking patients about their experience of T2D and what a cure for T2D, if there is one, would look like to them. The rest of the survey consisted of nine 5‐point Likert‐scale questions (e.g. 1 = strongly disagree, 5‐strongly agree). Interviews were audio recorded in the participant's private office. Survey responses and interview audio files were stored securely in a locked and encrypted Dropbox database.

### 2.3 Data analysis

Patient survey responses were thematically analysed for perspectives on the characteristics, outlook and influences on the concept of cure in T2D. All interview audio files were imported into Sonix and transcribed using Sonix's artificial intelligence programme. Once the transcription was complete, researcher David Zhao read the transcript while listening to the recording to correct spelling and grammatical errors, insert punctuation and anonymize the transcriptions. Interviewees were given the opportunity to review their interview transcripts and make corrections. Transcripts were thematically analysed by coding for topics, issues, similarities and differences revealed through the participants' narratives and interpreted by the researcher to understand the perspectives of cure in T2D in the professional epistemology. For example, perspectives were categorized on the appropriateness of using the word cure in T2D as well as their justifications.

## 3 RESULTS

### 3.1 Study population

Sixteen patients filled out surveys at the Kovler Diabetes Center. Of those, two surveys were incomplete, although the Likert items were completed in every survey. All 16 surveys are included in the analysis of the Likert items displayed in Table 1. A total of 10 interviews were conducted with various faculty involved in T2D patient care or research. Among these faculty were six practicing endocrinologists, one basic research scientist, two bariatric surgeons and a director of a weight management programme.

**Table 1:** Results of Likert item questions from patient surveys

Question (all *n* = 16)	Strongly disagree (1)	Disagree (2)	Neither agree nor disagree (3)	Agree (4)	Strongly agree (5)
I am confident in my own ability to manage my T2D.	*n* = 0	2	5	7	2
	0%	12.5%	31.25%	43.75%	12.50%
I am confident that my diabetes is curable or will be curable.	1	2	5	6	2
	6.25%	12.5%	31.25%	37.5%	12.50%
I initially sought treatment to be cured of my diabetes.	2	0	2	7	5
	12.5%	0%	12.50%	43.75%	31.25%
My family and friends have expressed their hope that I will be cured.	2	0	1	6	7
	12.5%	0%	6.25%	37.5%	43.75%
I am confident in my healthcare provider's ability to manage my diabetes.	0	0	2	4	10
	0%	0%	12.5%	25%	62.5%
I trust my healthcare provider's advice.	0	0	0	6	10
	0%	0%	0%	37.5%	62.5%
My healthcare provider has told me or implied that my diabetes is curable.	2	1	7	3	3
	12.5%	6.25%	43.75%	18.75%	18.75%
My overall well‐being would significantly improve if my type 2 diabetes is cured.	0	0	3	5	8
	0%	0%	18.75%	31.25%	50%

### 3.2 Patient perspectives on cure in type 2 diabetes

A total of 81% of patients viewed that their life would significantly improve if they were cured of T2D. About 75% of patients agreed or strongly agreed that they initially sought treatment to be cured of their T2D, which is in concordance with high agreement that a cure improving overall well‐being (Table 1). However, only about half of the participants were actually confident that their T2D would be cured, or were confident about managing it. The same two patients who strongly disagreed with seeking cure initially also strongly disagreed on the statement that their family and friends have expressed hope of cure for them, otherwise positive input from family and friends on the curability of a patient's T2D is common. A slight majority of patients were ambivalent about their knowledgeability of the literature on T2D treatment. About 100% of respondents agreed or strongly agreed with the statement ‘I trust my healthcare provider's advice’, although less than half the patients recall their provider ever expressing that their T2D could be cured departure from the initial expectation of seeking treatment to be cured.

Patient participants wrote about discrete manifestations of cure in two categories, a lifestyle cure and a pharmacological cure (Table 2). Some patients equate cure to lifestyle management, notably independent of visiting the doctor and citing ownership over maintaining a regimen of diabetic control. Patients' conceptions of cure tend to use the language of freedom. When patients were asked what cure means in comparison to management, the distinction between the two was freedom:

‐ Well overall my goal is to get off insulin and medicine overall. Having a cure would be great!
‐ It's having piece of mind that you don't have to worry about your sugars.
‐ Being able to be free of pills and/or shots would be the best.
‐ With management, you have to do what you have to do whether you like it or not.
‐ Cure would be great so I can eat what I want.
‐ To be able to eat foods that I like.
‐ I would be able to eat anything as often as I wanted to eat.

**Table 2:** Patient perspectives on the aesthetics of type 2 diabetes cure

**Lifestyle cure**	I would be able to eat anything as often as I wanted to eat. Just eating right, exercising.
	No doctor's appointment, medication regimen, watching food intake, monitoring of blood glucose. Losing weight has a lot to do with it.
	You got to eat the right type of food to help that.
**Pharmacological cure**	Cure means that I don't need all the drugs.
	Being able to maintain a healthy blood sugar level like healthy people with a one time ‘miracle’ medication.
	Pill form.
	There's got to be different types of medicine.
	Stem cells.
	A shot or even a patch.

Cure as freedom means changing from rigid to flexible restraint and eventually no restraint. The conception of a pharmacological‐based cure has two formulations based on the patient surveys: a cure that is a robust, one‐off pharmacological intervention, or a cure meaning the absence of all pharmaceuticals. Both suggest that current T2D treatment is burdensome.

### 3.3 Professional perspectives on cure in type 2 diabetes

Table 3 is a summary table of quotations regarding theme 1, perspectives of cure in the professional domain, from each interviewee emphasising where they stand on the curability of T2D. In the context of T2D treatment discussion, two interviewees found it appropriate to use cure in the context of T2D, and four interviewees were against the idea. The other four interviewees who were ambivalent justified their uncertainty with the possibility that a different term may be more appropriate to use rather than cure, such as remission or delay. There is also evidence of interdepartmental discussion of cure, where a bariatric surgeon cited the opinions of his endocrinology colleagues and how they have shaped his perspective about cure in T2D.

**Table 3:** Summary of T2D expert views of using the word cure in the context of T2D

Abstain	Ambivalent	Appropriate
‘Although I think one could argue meanings of the word cure and then have a difference of opinion, I generally lean towards do not use the word cure when I'm talking to patients’.	‘I would say that it's pretty hard to imagine that type 2 diabetes per se can be cured, but I think it's probably more reasonable to imagine that type 2 can go into remission’.	‘I might use the word “we can cure your diabetes” meaning we can remove that diagnosis from your problem list if we can maintain good blood sugar without medicines’.
‘I would never tell someone that their diabetes was cured…I say it can make your diabetes a lot better and make you free of medication…my bias is cure means for the rest of your life and that's why I never say that’.	‘When it comes to the language of cure, I tried to talk more about control, and then preventing and delaying complications’.	‘In my opinion, it's the weight loss that's really required if you want to cure the diabetes’.
‘I would not talk about cure for type 2 diabetes. It's unfortunate that over time a patient is thought to lose 60% or more of the beta cells. So some of the damage has already been done that I say, I don't expect the patient to come off for continuing the medication for diabetes’.	‘The word cure I think that we as surgeons tended to use it extensively in the context of the comorbidity remission after obesity surgery…But I do think that we have learned from our endocrinology colleagues as well seeing results over time that there can be some degree of a recurrence. And so I think that we're a little bit more careful about using the word cure and really thinking more about remission’.	
‘I'm a purist when it comes to a cure and I don't see any of these things [type 2 diabetes drugs] as being cures. I see them as being delays or temporary fixes or very good treatments’.	‘It's just interesting how physicians have moved away from the word cure…you're in a place where we can't detect that you have anything. Or you're in a place where you've resolved your diabetes in the context of you're not on any drugs and your glucose is normal…Maybe that is cure’.	

Remission is an important term that takes into account uncertainty in T2D due to multiple factors. Interviewed healthcare practitioners acknowledge the ideal of cure as a permanent resolution. One endocrinologist stipulated that if clinicians use cure for T2D, they ‘have to emphasize the fact that maybe it's not permanent…when I think about cure, I always think of it as a permanent thing or something that's likely to be’. Many interviewees did not find cure to be appropriate to promise in patient interactions (theme 3) for various reasons:

In my patients who have been recently diagnosed with type two diabetes, I use the word remission and the understanding that *type two diabetes is a genetic disease and we're absolutely not changing people's genes*. (italics for emphasis).

I think that when you say cured it makes it sound like there's like no underlying problem to begin with. And I feel like a lot of these patients have like this underlying whatever *genetic defect or predisposition for their pancreas not to be able to meet demand if you stress it out again*.

I think that the *environment being relatively toxic* for individuals who have a propensity to develop type two diabetes, a substantial amount of the control is not really there.

The general consensus among these participants is the thought that there is always a risk of the disease or contributing factors, like obesity, returning or persisting. These may be outside the control of either the patient or clinician. An endocrinologist summarizes the potential to misrepresent any expectation of cure: ‘What I'm concerned about is that if we use the word cure, we're going to think that individual then will not expect to at anytime in his or her life have high blood sugar from type two diabetes’. The fear of many providers is that using the word cure from the professional perspective will suggest to patients that they will have to do less in terms of managing their T2D. This may be why healthcare practitioners often prefer remission, because it is less ambiguous than cure at communicating the uncertainty and risk in T2D prognosis. With remission, a patient may still have to watch their weight to minimize the chances of recurrence, but with cure a patient might think that no matter their weight control behaviour, their T2D will never return. A contrary thought is that the term cure can be more useful than remission because cure can serve as a signal of hope for patients. A bariatric surgeon acknowledges that ‘from a clinical standpoint, remission is a more accurate terminology, right. But I do think that the lines that we use to communicate to patients and the context of hope and change is really important too’.

### 3.4 Notable differences on perspectives of cure

Patient conceived lifestyle and pharmacological T2D cures are at odds with expert beliefs about how the lifestyle and pharmacological aspects of T2D treatment interact. Responses regarding to the second theme of how cure could be achieved acknowledge the necessity to modify the molecular pathophysiology of T2D. From the perspective of an interviewed bariatric surgeon on pharmacological interventions, to cure T2D, the treatment has ‘to change…the complexity of something like eating behaviours and, and glucose homoeostasis, it's not going to be a single pathway, a single drug sort of response because there's so many different inputs’. Another endocrinologist explains that a single miracle drug is unlikely to come to fruition because even newer drugs are not addressing the underlying causes of T2D; they are not ‘disease modifying agents’. Current treatments do not address the underlying cause of T2D but rather focus on symptom management and maintaining blood glucose homoeostasis.

In terms of the aesthetics of cure, or the imagined modality of cure, patients have a tendency to envision cure being in the form of a pill, or a lifestyle change, or even shots or a patch (Table 2). Interestingly, patients did not mention surgery as an image of cure. In contrast to pharmacological interventions, a bariatric surgeon mentioned that ‘other than bariatric surgery, there's really no effective drug out there that will promote weight loss without causing side effects…from my point of view, it's the weight loss that's really required if you want to cure the diabetes’. Bariatric surgery can not only be an alternative to medications, but a possible pathway to get off of them, ‘if one means can it be such that people can get off all medications and have essentially normal blood sugars, then in some patients, bariatric surgery might cure type 2 diabetes’. Even then, surgery would be a pathway to come off pharmacological dependence, a harbinger for cure to patients.

## 4 DISCUSSION

T2D patients and professionals have different perspectives on the curability of and the characteristics of cure in T2D. Patients base their perspectives from their experiences with and without T2D, the suffering and hope engendered by the process of contending with T2D. Clinicians and researchers tend to draw from the perspective of scientific medicine to deliver treatment within ethical and technical boundaries. As a result, there is heterogeneity in the conception of cure in chronic disease. Cure may be defined clinically, with varying thresholds and tolerances, as well as in terms of lifestyle or pharmacological independence. To complicate matters, the wide use of alternative terms to cure (remission, reversal, delay and post‐diabetes) among interviewees and in the literature without clear distinctions between each further reflects the great uncertainty in T2D outcomes. There is also variation in a T2D patient's ability to adhere to interventions, background and underlying risks that must be considered.

Perspectives of cure in T2D are dependent on perceived experience, which shape the concept of cure in lay epistemology in response to the uncertainty of the disease's outcome. For patients, cure is simultaneously nostalgic, while also hopeful for the future with the return of freedom and normalcy. Canguilhem referred to this as ‘the general and persistent tendency to conceive the cure as an end to a disturbance and a return to an anterior order’.^19^ For T2D patients, the cure in the future is often in reference to a past state, since many T2D patients remember a period of normalcy before they were diagnosed. Cure is ideally something that ends an impediment on health and freedom that is not only borne from the condition itself but also its chronic treatment, such as the pain of insulin injections.^14^ From the patient perspective, this may not have much to do with the clinical markers (e.g., insulin sensitivity, blood glucose and Hba1c) of T2D, but more so the functional markers (e.g., diet, ability to perform simple tasks and body image). Thus, the concept of cure may serve as a strong motivating factor to reclaim functional health and independence. However, not every patient has the ability to harness lifestyle management into a potential cure, and many of the interviewed providers believed lifestyle choices cannot overcome permanent underlying risk factors. These barriers include behavioural or personal barriers to adherence,^22^, ^23^ social barriers,^24^ economic barriers^25^ and cultural/religious barriers.^26^ This is when they reach for support from T2D professionals and envision pharmacological cures.

The concept of cure is linked to clinical uncertainty^27^ in the professional epistemology due to the high number of variables affecting the aetiology and prognosis of T2D. Uncertainty prompts consideration of alternatives to cure in T2D, such as remission, management and control in the professional domain. These terms capture the essence of T2D as a chronic condition and the conservatism in labelling a cure too soon. On the other hand, for patients, cure is linked to uncertainties in relative lifestyle quality in patients more so than clinical uncertainty. The patient‐centred aspect of T2D care puts into question whether a cure necessarily requires determination from a medical professional. Canguilhem explains a difference in conception of cure between medical professionals and patients^19^:

While to the traditional medical eye a cure was the effect of a treatment of the cause and functioned to sanction the validity of a diagnosis and the ensuing prescription—hence of the doctor's own worth—to the psychoanalytic eye, a cure became the sign of the patient's rediscovered capacity finally to be done with his own difficulties. A cure was no longer commanded externally; it became an initiative recuperated by the patient.

Since the management of weight, blood sugar levels and even medication is primarily the responsibility of the patient, do any of the clinical indicators of diabetes matter if the patient simply feels cured, whatever metric they decide to substantiate that on? It may be that the role of the healthcare professional is to mediate clinical realities with patient ideals about T2D care, not to determine whether a patient is cured of T2D.

An example of bridging expectations and perspectives about the concept of cure in T2D could be a discussion on the role of bariatric surgery in T2D treatment. A study found that few patients with T2D viewed bariatric surgery as a safe treatment for T2D and there was no mention of bariatric surgery among patients in this study.^28^ Yet, there are T2D experts, including in this study, who believe bariatric surgery can have a considerable impact on T2D, and even cure it.^28^, ^29^ A cure for T2D by bariatric surgery may be considered after a certain amount of time spent in remission. But it is worth noting that 35% of T2D patients who achieved complete remission within 5 years of bariatric surgery also redevelop T2D in a later point within 5 years of surgery, and few studies track patients beyond 5 years for which the course is more uncertain.^30^ To reconcile cure and remission requires an elicitation of the patient's values. Is it meaningful to come off of T2D medication following successful bariatric surgery, and if so for how long? How much relative freedom in lifestyle management should be gained after bariatric surgery to constitute independence from the limitations of T2D? In some cases, remission may be equated to cure.

The role of a medical professional when dealing in the mutual conceptualisation of cure with their patients is to balance communicating clinical realities—such as underlying risk, complications and treatment guidelines—to the patient, while also acknowledging patient goals and values. This requires a shared decision making where the provider elicits the patient's explanatory model of their own illness, their T2D ontology, in to understand their beliefs and personal narratives about why they have T2D and how they may overcome it.^31^ This may shift a provider's strategy from emphasising evidence‐based care, such as utilising a patient's labs and history to manage their diabetes, to person‐centred care, using a patient's perspective and the resources they have to empower them to find their own cure. The patient perspective is thus brought up to the same level as the professional perspective.

Surveyed patients had high trust in their providers, both in their words of advice and ability to treat their T2D. This trust is important as it most likely moderates the high initial patient expectation for cure and the reluctance of providers to talk about a cure for T2D. In another study in Scotland, T2D patients found that the perceptions about T2D can be mutually informing during interactions with health services.^32^ While the hopeful element of cure can be useful, false reassurance can be misguiding. Medical anthropologist Mary‐Jo DelVecchio Good also warns, ‘those who suffer serious illness become particularly susceptible to hope engendered by the cultural power of the medical imagination’.^33^ It is unethical to promise a cure without proper discussion between all stakeholders of what is meant by cure based on medical realities. Hope is important, but it should not be at the expense of trust and clear communication. Key to this communication would be, as Donald Light argued, avoiding overconfidence in medical knowledge and disregard towards patient‐centred ideas of health and illness.^34^ The primary goal is not to achieve a cure as understood by any particular epistemology, but to reach a mutual understanding of cure through trust and communication. Then can a decision be made about whether that concept of cure fits with a realistic outcome.

This study has several limitations. First, participants were recruited from one academic medical centre in the Midwest. Although multiple specialities were represented, all the patients surveyed were recruited at the Kovler Diabetes Center at the University of Chicago Medical Center, a clinic in an urban setting. Regional differences may account for different perceptions, for example, through different standard of care practices for T2D in different medical centres. Second, demographic data such as age, gender, education background and ethnicity were not collected. It is possible that differences in these backgrounds could explain the development of participant perspectives. Additionally, clinical indicators of patients' T2D describing the severity and duration of their illness was not collected. How long and how much a patient has suffered from T2D may impact their ideas about cure. Given that the patients were recruited at a diabetes clinic in a tertiary healthcare institution, rather than a primary care clinic, it is likely that these patients have been referred for complex management of their diabetes and may have more severe presentations of T2D. The third limitation was the lack of interview data from patients. The decision to give patients a brief survey instead of an interview was made in the interest of a patient's privacy and time since they were recruited following an outpatient endocrinology appointment. However, interviews could have added additional richness to the patient perspective. Finally, the small sample size of patients and experts who participated means these findings are preliminary and exploratory.

In conclusion, providers are in the position to empower T2D patients towards obtaining their goals, and to frame the problem as well as the solution. T2D professionals can help manage expectations in the management and outcomes of T2D through education and communication. However, cure is not always explicitly discussed between providers and patients. Clinicians may even avoid the use of the word cure entirely, but patients will often come in with a preconceived expectation about the image of cure. The goal should not be to avoid the topic of cure in T2D altogether. However, to avoid potentially dangerous confusion, it is paramount that communication is focused on understanding what others think about cure in addition to one's own assumptions and perspective, identifying common conceptions, mediating differences and establishing roles to achieve the mutually constructed goal, which may not necessarily be a cure. Talking about cure does not always result in cure, but it may result in other positive outcomes, such as finding what it means to thrive with a chronic condition like T2D. The ideal cure may invoke a defining, straightforward goal of medicine, but the interpretations of cure in the context of a chronic disease such as T2D reveal that cure is not a deterministic or concrete goal, but a concept that can be leveraged to the benefit of treatment.

## CONFLICT OF INTERESTS

The author declare no conflict of interest to disclose.

## Supporting information

Supporting information.

## Data Availability

The data that support the findings of this study are available from the corresponding author upon reasonable request.
