# Supplementary material for: Goals of cure: Perspectives on the concept of cure in type 2 diabetes
Source: J Eval Clin Pract. 2022 Feb 12;28(3):445–53. doi: 10.1111/jep.13666 (PMC9303532; doi:10.1111/jep.13666)
Supplement: Supplementary file 1 — Supporting information. [file JEP-28-445-s001.docx]

Cure Interview Questions for T2D experts (asked in order):

The current perception of cure in T2D in the professional domain

1. Have you or any of your colleagues expressed that type 2 diabetes is curable?
2. Do you think type 2 diabetes will be curable in the near future?
3. What would a cure for type 2 diabetes look like?

How cure could be achieved

1. What are the goals of your research (or care)?
2. Can research alone cure type 2 diabetes?

Cure in patient interactions

1. How do you treat type 2 diabetes patients? What considerations go into treatment plans?
2. Is it ethical to tell a patient now that is it possible for their diabetes to be cured?
3. Have you ever been asked by a patient to cure their condition? What do you tell them?
4. What are barriers to cure? (financial, social, technical, cultural)
